# Charge storage characteristics of Au nanocrystal memory improved by the oxygen vacancy-reduced HfO_2_ blocking layer

**DOI:** 10.1186/1556-276X-8-368

**Published:** 2013-08-28

**Authors:** Ruifan Tang, Kai Huang, Hongkai Lai, Cheng Li, Zhiming Wu, Junyong Kang

**Affiliations:** 1 Semiconductor Photonics Research Center, Department of Physics, Xiamen University, Xiamen 361005, China

**Keywords:** Memory performance, Oxygen deficiency, Annealing, Non-volatile memory, 85.35.-p, 61.72.Cc

## Abstract

This study characterizes the charge storage characteristics of metal/HfO_2_/Au nanocrystals (NCs)/SiO_2_/Si and significantly improves memory performance and retention time by annealing the HfO_2_ blocking layer in O_2_ ambient at 400°C. Experimental evidence shows that the underlying mechanism can be effectively applied to reduce oxygen vacancy and suppress unwanted electron trap-assisted tunneling. A memory window of 1 V at an applied sweeping voltage of ±2 V is also shown. The low program/erase voltage (±2 V) and the promising retention performances indicate the potential application of NCs in low-voltage, non-volatile memory devices.

## Background

Nanocrystal (NC) floating gate memory devices have recently attracted much attention as a strong candidate for non-volatile memories given their scalability, fast write/erase speeds, low operating voltages, and long retention times [1-4]. Numerous attempts have been made to develop non-volatile memory devices using metal NCs, such as Ni [5], Au [6], Ir [7], and Pt [8], because metal NCs have a higher density of states around the Fermi level, a wider range of available work functions, and smaller energy perturbation compared with their semiconductor counterparts [9]. Further improvement in memory performance can be achieved through the integration of metal NCs with high-*κ* dielectric materials, such as HfO_2_[10] and Al_2_O_3_[11]. The use of high-*κ* dielectric materials as blocking layers decreases the electric field at the top dielectric and program/erase (P/E) voltages, which also supports the demand for small effective oxide thickness [12]. Au NCs with high work functions (5.1 eV) enable the creation of a deep potential well to trap charge carriers, such as HfO_2_, with high dielectric constants (20 to 25) and relatively high barrier heights (−5.7 eV). The structure of metal/HfO_2_/Au NCs/SiO_2_/Si shows a strong potential for application in non-volatile memory devices [13,14].

Metal/HfO_2_/Au NCs/SiO_2_/Si is fabricated in this study. The capacitance-voltage (*C*-*V*) characteristics show that the main storage consists of holes. However, electron trapping is seldom achieved because of the HfO_2_ blocking layer. X-ray photoelectron spectroscopy (XPS) confirms that the oxygen deficiency within the HfO_2_ layer is caused by the presence of Hf-Hf bonding. The energy band diagram shows that electrons trapped in the NCs tend to leak into the gate electrode through trap-assisted tunneling, which is supported by the oxygen vacancy-related levels during programming. However, Hf-Hf bonding disappears after HfO_2_ is annealed at 400°C for 10 min in O_2_ ambient. The structure of metal/HfO_2_ (as-annealed)/Au NCs/SiO_2_/Si shows that both electrons and holes are stored. Given their memory window of 1 V at an applied sweeping voltage of ±2 V, low P/E voltage (±2 V), and promising retention performances, low-voltage NC memories have a strong potential for application in non-volatile memory devices.

## Methods

A metal/HfO_2_/Au NCs/SiO_2_/Si (A_1_) structure was fabricated. P-type Si with a doping level of 8.33 × 10^17^ cm^−3^ was used as a substrate. A 3-nm-thick thermal SiO_2_ oxide was fabricated using a rapid thermal annealing (RTA) device after pre-gate cleaning. An Au film with a thickness of approximately 1 nm was sputtered using SCD005 (Balzers Union, Balzers, Liechtenstein) with a sputtering time of 2 s. The sample was then annealed in N_2_ ambient using the RTA device. Annealing was performed at 600°C for 10 s to form Au NCs. A 30-nm HfO_2_ film deposited by the electron beam (E-beam) evaporation system with a base pressure of 3.6 × 10^−6^ Torr served as the blocking layer. After depositing the TaN/Al metal gate electrode with thicknesses of 50/300 nm and the Cr/Au bottom electrode with thicknesses of 20/200 nm through magnetron sputtering, the capacitive structure of the NC memory device was finally completed. Metal/HfO_2_/SiO_2_/Si (A_2_), metal/SiO_2_/Au NCs/SiO_2_/Si (A_3_), and metal/HfO_2_ (PDA)/Au NCs/SiO_2_/Si (A_4_) were fabricated using the same process, with the exception of a 20-nm SiO_2_ film deposition using the E-beam for sample A_3_ and the annealing of HfO_2_ after deposition at 400°C for 10 min in the O_2_ ambient for sample A_4_. XPS with a 1,486.6-eV Al Kα source was used to obtain composition information about the as-deposited and annealed HfO_2_ film. The electrical characteristics of the NC memory devices were measured in the parallel mode using a Keithley 4200 semiconductor characterization system (Cleveland, OH, USA) and a Keithley 590 C-V analyzer at room temperature.

## Results and discussion

**Figure 1:**
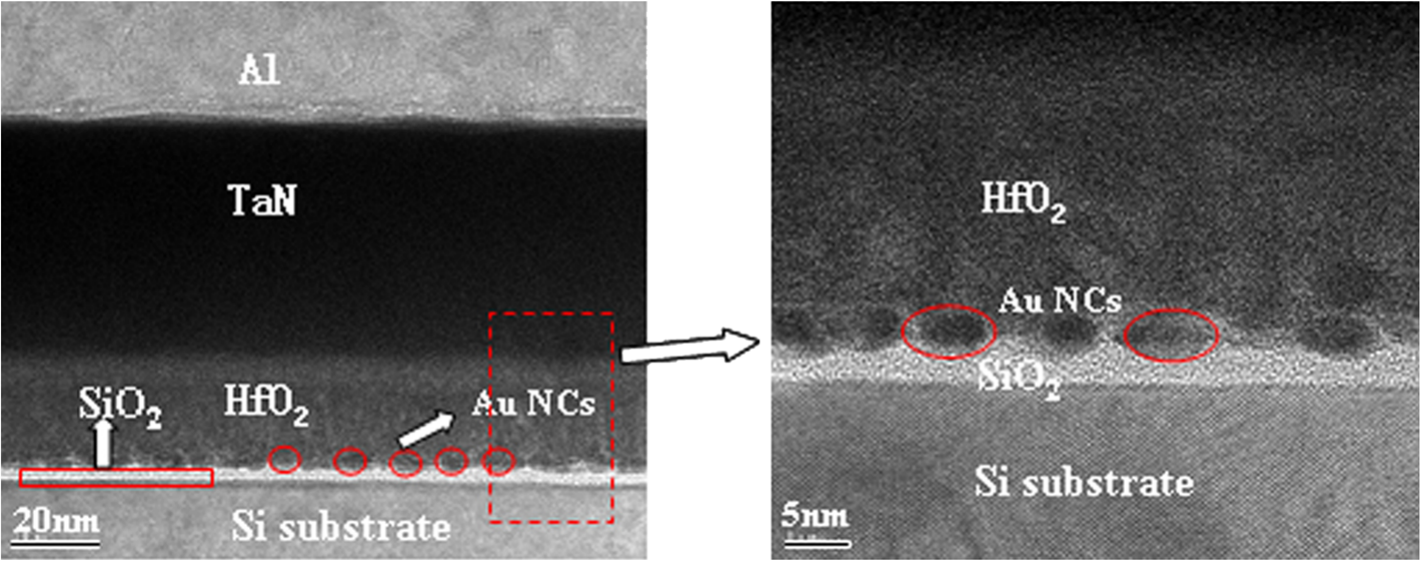
**Cross-sectional HRTEM micrograph of sample A**_**1**_**.**

**Figure 2:**
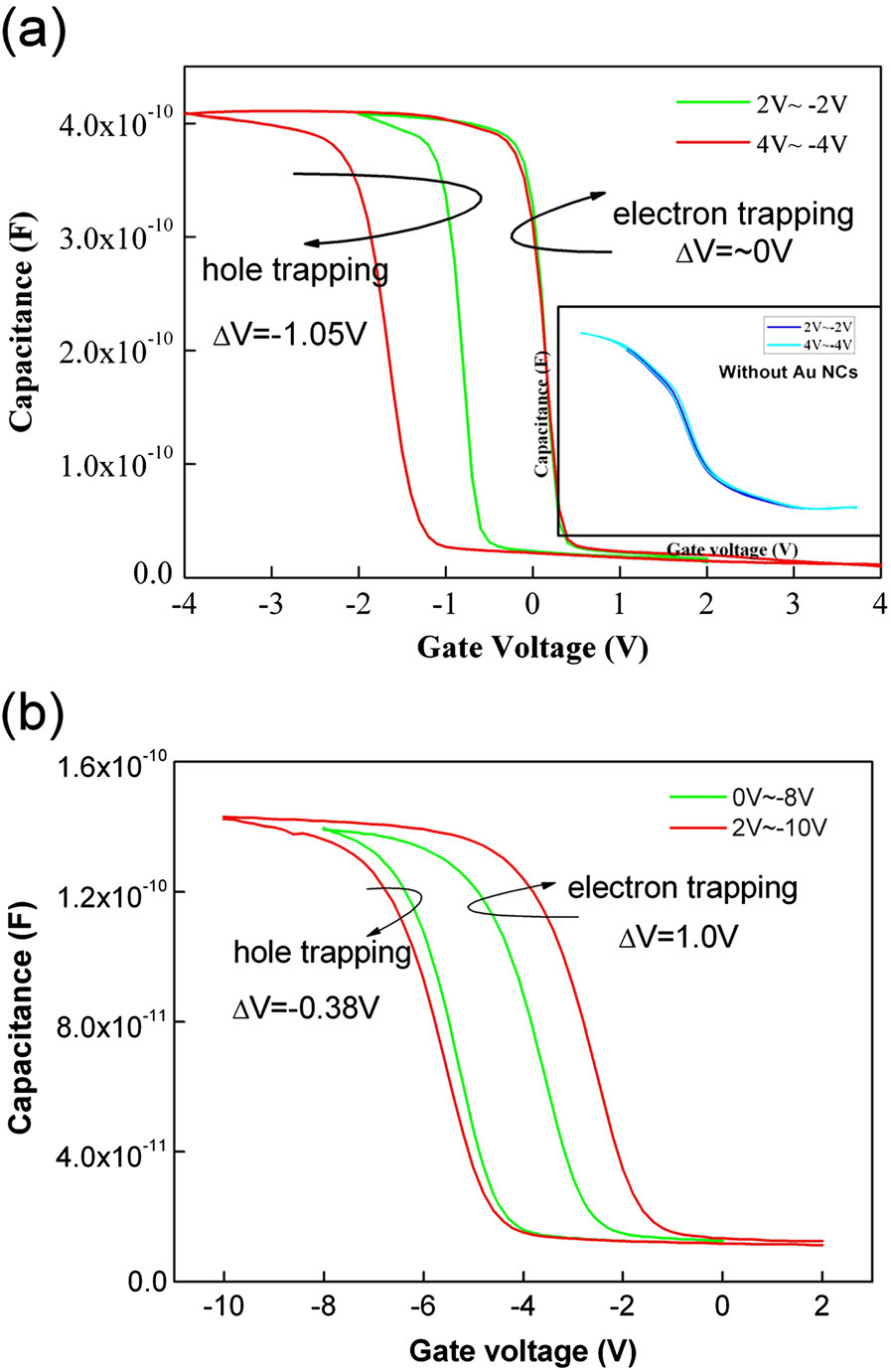
***C*-*V *hysteresis of sample A**_**1 **_**(a) and sample A**_**3 **_**(b).** The inset plot in **(a)** shows the *C*-*V* curves of sample A_2_.

Figure 1 shows the cross-sectional high-resolution transmission electron microscopy (HRTEM) micrograph of the A_1_ device. The Au NCs formed on the 3-nm thermal SiO_2_ are covered with a 30-nm HfO_2_ layer. The NC density is approximately 8 × 10^11^ cm^−2^, wherein the size is mainly distributed from 6 to 8 nm. The charging properties are described from the *C*-*V* measurements at 1 MHz with a step of 0.1 V/s for A_1_ (Figure 2a). Double *C*-*V* sweeps are performed with voltage sweeps from inversion to accumulation, i.e., from positive to negative bias and back to inversion to give prominence to the charge trapping in the Au NCs. Electron and hole trapping in the NCs are enabled by the positive and negative biases, respectively. The positive flat band voltage shifts (Δ*V*) correspond to an increase in electron trapping, whereas the negative Δ*V* corresponds to the increase in hole trapping given the increasing sweep voltage range. Figure 2a shows that the negative Δ*V* is about 1.05 V, whereas the positive Δ*V* is close to 0, which indicates that no additional electrons can be trapped with the increase in the sweep range. The inset plot in Figure 2a shows the *C*-*V* curves of sample A_2_. Sample A_2_ showed no apparent hysteresis loop both at the ±2 and ±4 V bias sweep, indicating that a charging effect only occurs with Au NCs. The electron’s energy barrier of 3.2 eV between Si and SiO_2_ is known to be much less than that of the hole (4.7 eV). Electron tunneling is expected to be easier than hole tunneling. However, the *C*-*V* characteristic shown here indicates that electron trapping is more difficult than hole trapping. One possible reason is because the electrons trapped in the Au NCs leak back to the substrate and result in lessened electron trapping, which is similar to previous reports [15]. In previous reports, a band offset exists at the valence band between Ge and Si. Holes can be trapped in Ge_1 − *x*_Si_*x*_/Si heteronanocrystals, whereas electrons tunnel back to the substrate directly through the ultrathin tunnel oxide. However, these reports are inconsistent with our experiments because no additional barrier layer for holes exists in our experiments; thus, lessened electron trapping cannot be attributed to electron loss in thin tunnel oxide.

**Figure 3:**
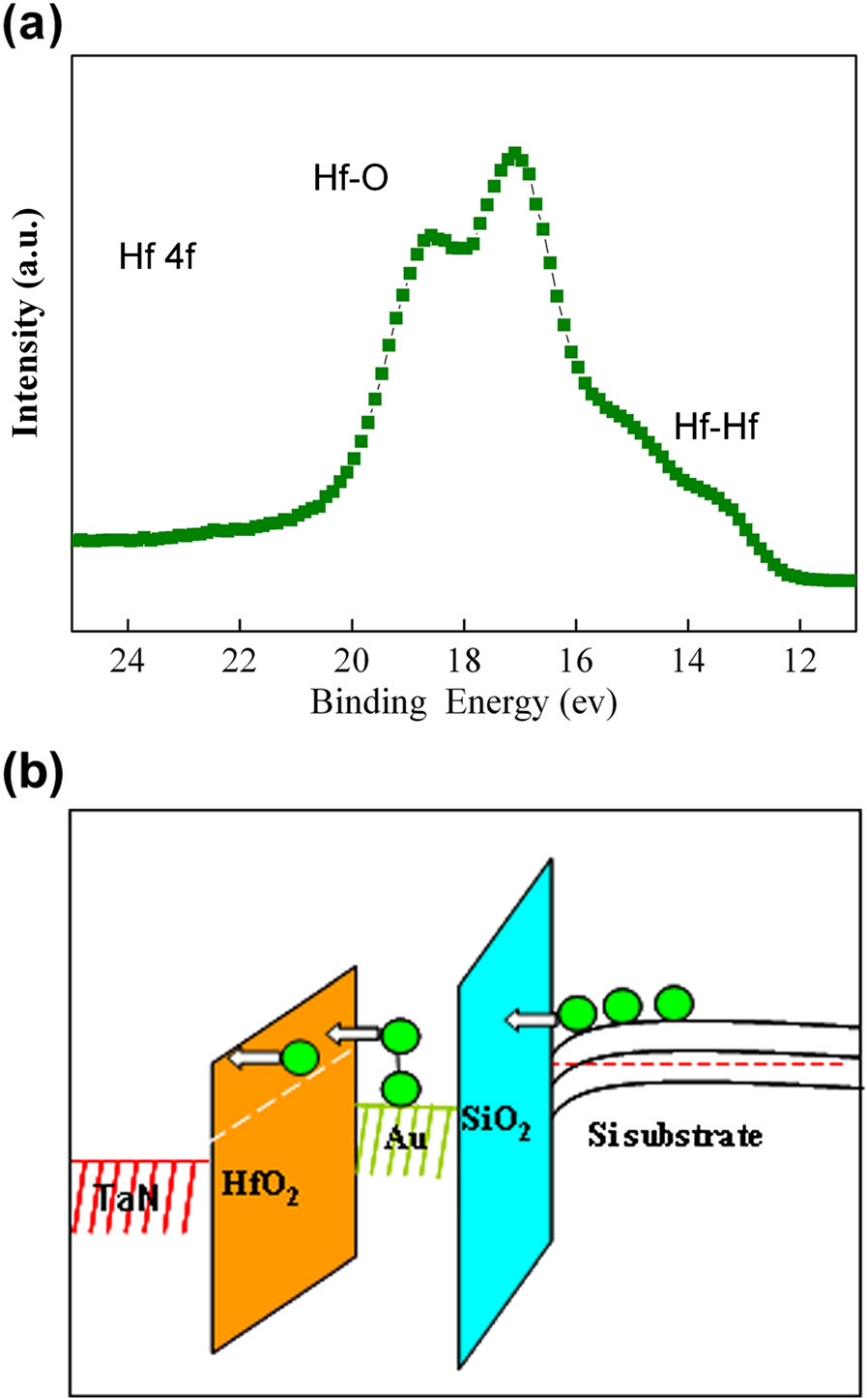
**XPS spectra and energy band diagram. (a)** Hf 4*f* core-level XPS spectra of as-deposited HfO_2_ film and **(b)** energy band diagram of sample A_1_ during programming.

Another possible mechanism leading to electron injection from the inverted substrate into the Au NCs during programming is the positive gate bias. Electrons are emitted from the NCs, which cross the HfO_2_ blocking layer to the gate electrode [16]. Sample A_3_ is fabricated with SiO_2_ as the blocking layer to investigate the effect of HfO_2_ and the possible mechanism. The control oxide thickness of SiO_2_ in sample A_3_ is noted to be about 20 nm; to lessen the electric field differences between samples A_1_ and A_3_ during the sweep process, the sweeps are performed from −8 to 0 V and −10 to 2 V. Figure 2b shows the *C*-*V* hysteresis curves for A_3_ with sweep ranges of −8 to 0 V and −10 to 2 V. The positive Δ*V* is approximately 1 V and is greater than the negative Δ*V* (0.38 V) with the increase in sweep range. A high positive Δ*V* value indicates that both electrons and holes can be stored in NCs. Electron trapping is also easier than hole trapping, which is consistent with previously reported theories and results [17,18]. Therefore, the asymmetric *C*-*V* hysteresis curve of A_1_ is reasonably caused by the HfO_2_ blocking layer. The HfO_2_ films prepared using different growth methods have different microstructures and properties [19]. XPS measurements are performed using our E-beam device to investigate the composition information of the as-deposited HfO_2_ film. About 2 nm of the sample top layer was removed using Ar ion bombardment to remove surface contaminants. Figure 3a shows the two peaks at 17.1 and 18.6 eV, which correspond to the Hf 4*f* and Hf 4*f* peaks from HfO_2_. Small but noticeable shoulders at the lower binding energy side of the main peak were also observed, which can be attributed to Hf-Hf bonding and indicate the existence of oxygen vacancy within the HfO_2_ film [20]. Oxygen vacancy reportedly results in oxygen vacancy-related levels within the bandgap [21]. Takeuchi et al. used spectroscopic ellipsometry to demonstrate the existence of shallow oxygen vacancy-related defects 1.2 eV below the HfO_2_ conduction band [22]. Given the existence of an oxygen vacancy-related level below the conduction band and the rise of electron potential because of electron trapping in the NCs [23], electrons trapped in Au NCs could possibly leak into the gate electrode through the trap-assisted tunneling method during the programming operation (Figure 3b). This method is similar to the multi-phonon-assisted tunneling model described in previous reports [24]. The trap-assisted tunneling effect may be responsible for the minimal electron storage.

**Figure 4:**
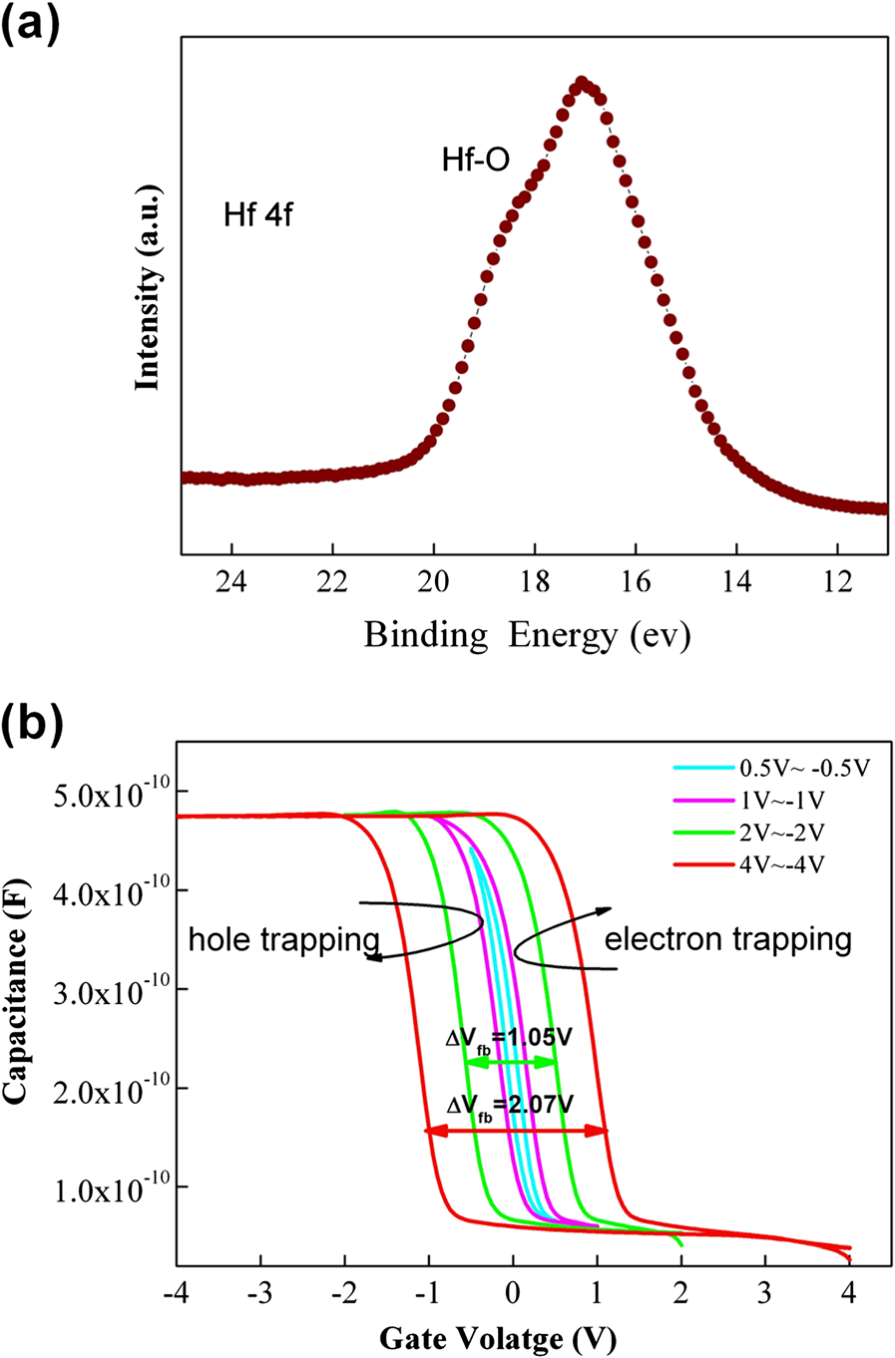
**XPS spectra and *C*-*V *hysteresis. (a)** Hf 4*f* core-level XPS spectra of as-annealed HfO_2_ film and **(b)***C*-*V* hysteresis of sample A_4_.

**Figure 5:**
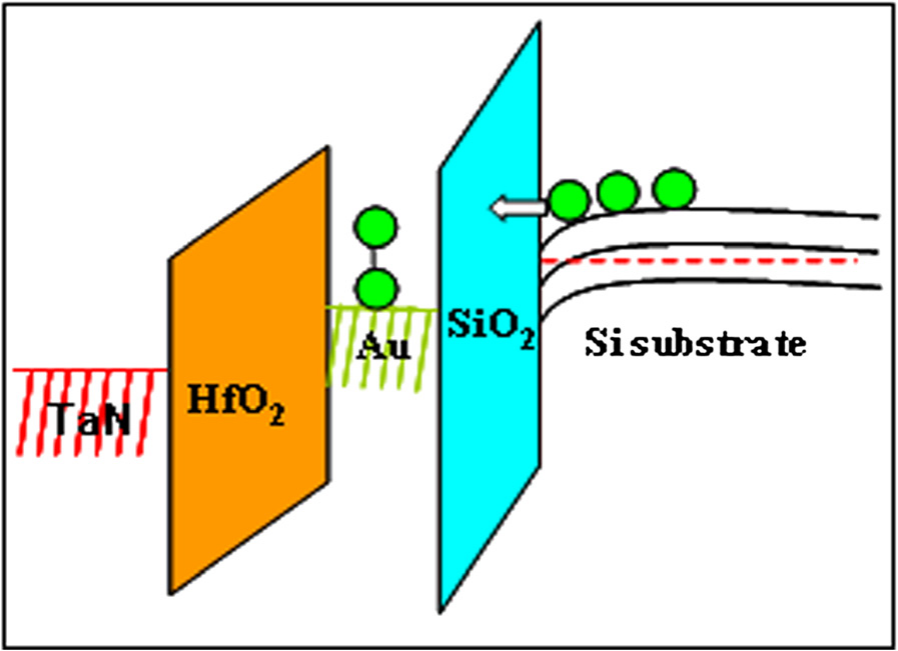
**Energy band diagram of sample A**_**1 **_**during programming.**

**Figure 6:**
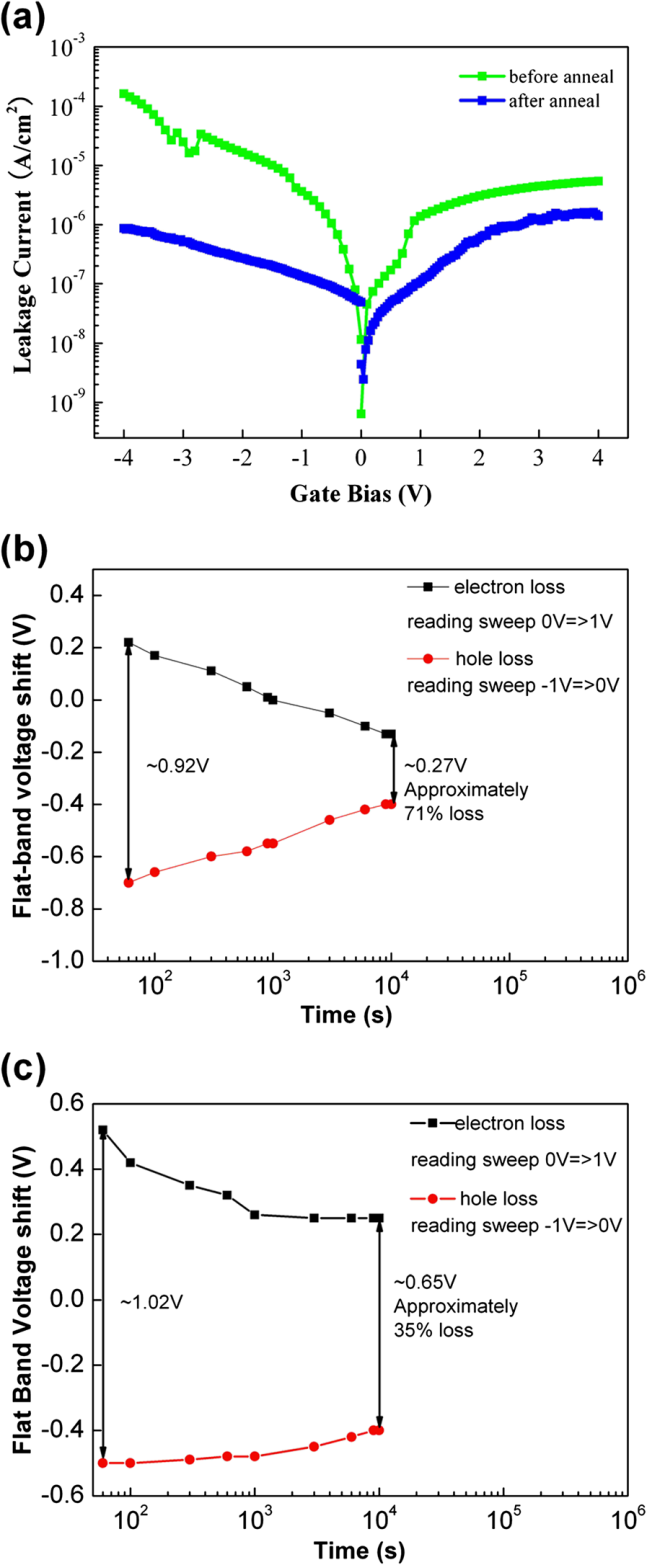
**Leakage currents and charge retention property. (a)** Comparison of the gate stack leakage currents of samples A_1_ and A_4_, and charge retention property of samples **(b)** A_1_ and **(c)** A_4_.

**Figure 7:**
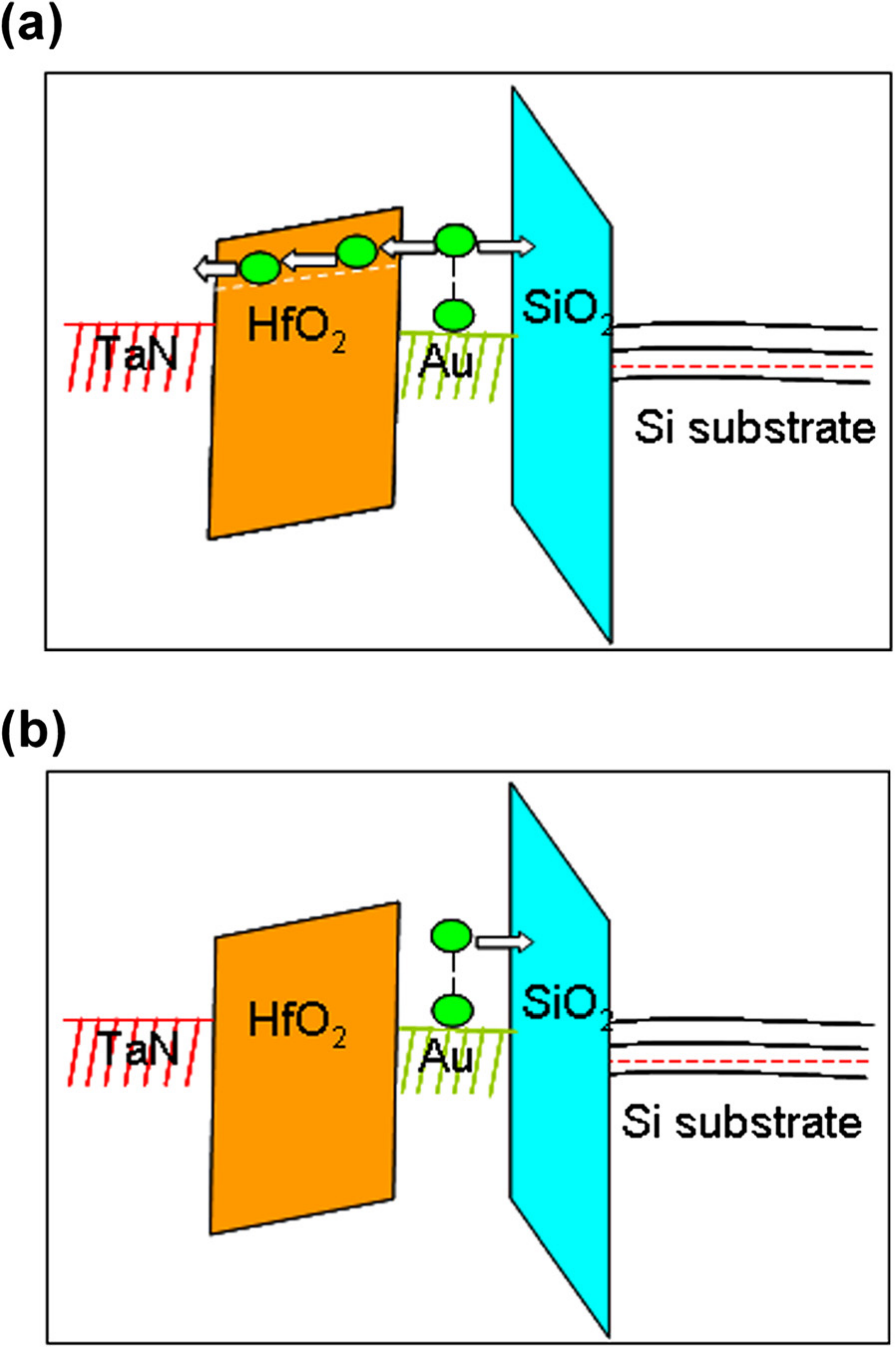
**Energy band diagram of samples (a) A**_**1 **_**and (b) A**_**4 **_**during retention.**

HfO_2_ was annealed after deposition at 400°C in O_2_ ambient to verify this assumption. XPS analysis was performed on the O_2_-annealed HfO_2_ film after 2 nm of the HfO_2_ top layer was removed by Ar ion bombardment to remove the surface contaminants. Figure 4a shows that no evidence of Hf-Hf bonding was observed, with the exception of the characteristic peak attributed to Hf-O bonds. This lack of evidence suggests that the annealing process can effectively reduce the oxygen vacancy of HfO_2_ films. Sample A_4_ was fabricated using the O_2_-annealed HfO_2_ as blocking layer. Figure 4b shows the *C*-*V* characteristics of A_4_. The positive Δ*V* is almost similar to the negative Δ*V* with the increase in the sweep voltage range, thereby indicating that both electrons and holes can be easily stored in the Au NCs. The ease of electron and hole storage is caused by the reduced oxygen vacancy levels and the suppressed unwanted electron trap-assisted tunneling performed during programming, which leads to electron storage (Figure 5). Electron storage can be confirmed further through a comparison of A_1_ and A_4_’s gate current characteristics. Figure 6a shows that sample A_4_, with an O_2_-annealed HfO_2_, shows lower leakage current density at all regimes of the gate voltage compared with sample A_1_, with an as-deposited HfO_2_. The lower leakage current indicates that the reduced oxygen vacancy-related levels suppress electron injection from both the substrate and gate given that the positive gate voltage corresponds to substrate injection and the negative gate voltage corresponds to gate injection. Figure 6b,c shows the retention properties of A_1_ and A_4_. The initial memory windows are 0.92 and 1.02 V for A_1_ and A_4_, respectively. The windows are followed using a suitable reading condition. The decayed charges for sample A_4_ with O_2_-annealed HfO_2_ were only 35% within a 10^4^-s span, which is much better than that of A_1_ (approximately 71% loss). The difference between the observed retention behavior of A_1_ and A_4_ could be explained by the energy band diagram, which is based on the existence of oxygen vacancy-related levels. Figure 7a shows that the electrons trapped in the Au NCs leak into the gate electrode through the HfO_2_ layer via electron tunneling to the oxygen vacancy-related level, as proposed in [24]; therefore, discharging easily occurs. However, the reduced oxygen-related levels in sample A_4_ HfO_2_ layer suppress the unwanted trap-assisted tunneling (Figure 7b); thus, electron loss rate is reduced.

**Figure 8:**
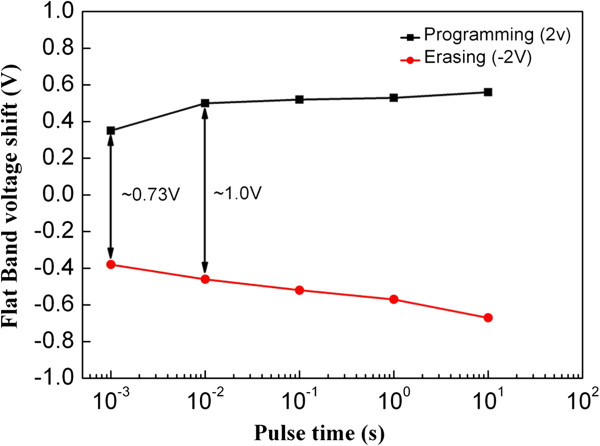
**P/E characteristics of sample A**_**4 **_**with as-annealed HfO**_**2 **_**for P/E voltage levels of +2/−2 V.**

A 1-V memory window was observed for A_4_ at the ±2-V sweep (Figure 8), which shows the potential to prepare a low-voltage NC memory. The P/E operation was also performed by applying ±2-V pulses to the gate electrode. Figure 8 shows that a 1-V memory window can be obtained at P/E times of 10/10 ms, which shows a sufficient memory window even at a ±2-V applied pulse voltage. Given the improvements in the retention performances (Figure 6c), sample A_4_ shows promise for application in low-voltage NC memory.

## Conclusions

Electrons trapped in Au NCs tend to tunnel into the gate electrode through the oxygen vacancy-related levels of the HfO_2_ blocking layer and tend to degrade memory performance because of the existence of oxygen vacancy. Annealing the HfO_2_ blocking layer at 400°C in O_2_ ambient decreases oxygen vacancy and suppresses unwanted electron trap-assisted tunneling. Given their memory window of 1 V at an applied sweeping voltage of ±2 V, low P/E voltage of ±2 V, and improved retention performances, low-voltage NC memories show promise for application in non-volatile memory devices.

## Abbreviations

E-beam: Electron beam; HRTEM: High-resolution transmission electron microscopy; NCs: Nanocrystals; P/E: Programming/erasing; RTA: Rapid thermal annealing; XPS: X-ray photoelectron spectroscopy.

## Competing interests

The authors declare that they have no competing interests.

## Authors’ contributions

RT carried out the experiments studied on the device fabrication and drafted the manuscript. KH designed the research programs and guided the experiment’s progress. HL, CL, ZW, and JK participated in the mechanism development. All authors read and approved the final manuscript.
